# Recurrent Appendicitis*Reported to the Louisville Clinical Society.

**Published:** 1897-11

**Authors:** William C. Dugan

**Affiliations:** Louisville, Ky.; Professor of the Principles and Practice of Surgery and Surgical Pathology in the Louisville Medical College, etc.


### RECURRENT APPENDICITIS.*

BY WILLIAM C. DUGAN, M. D., LOUISVILLE, KY.

Professor of the Principles and Practice of Surgery and Surgical Pathology in the Louisville Medical College, etc.

I recently had a case of recurrent appendicitis that is rather interesting. A young man had had four attacks previously and on Thursday night felt sick and consulted his physician. At supper time he was all right; after supper he began to feel badly and was seen by a physician at eight o'clock. He was told that he had better go to bed, that he had an attack of appendicitis, and that the doctor would see him again in the morning. Friday morning he was very much worse; operation advised to which the patient consented. I saw him Friday afternoon at three o'clock. At that time there was no distinct tumor, but the muscular rigidity was such that we could not manipulate the abdomen with any satisfaction. He was sent to the infirmary and at 4:30 o'clock in the afternoon we operated, twenty hours after the initial symptom of a recurrent attack. To our great surprise we found a large amount of pus had already formed at the site of the trouble; the omentum was several inches thick and matted down to everything. We had some difficulty in removing it. The young man had complained of a great deal of pain up under the costal cartilages; we found the omentum very firmly attached up in that region, and after some searching found a secondary abscess located there.

The points of interest in the case are: The rapid formation of pus, within twenty hours after the initial symptom; second, the importance of separating all the omentum at the time of the operation. If we had neglected this precaution we would have left the secondary abscess which I am satisfied would have so complicated matters as to have caused the young man's death. The operation was rather complicated; we removed all of the omentum. In the wall of the ecum there was a space as large as the palm of my hand which looked to be almost gangrenous. This was placed so it could be separated from the general cavity by gauze packing, and the gauze is still *intact*. The young man's pulse was 128 while on the operating table, but since the operation it has fallen to 70 and he is doing very well.

### DISCUSSION.

DR. LOUIS FRANK: Suppuration in most of these cases is due to the colon bacillus, and pus sometimes forms very rapidly. It is not unusual twenty-four or thirty-six hours after the onset of the trouble to find pus, also perforation or a gangre-

*Reported to the Louisville Clinical Society.

nous condition of the appendix. I have seen in the last ten days three cases of appendicitis. Dr. Vance saw one with me, in which operation was urged and refused. All three cases recovered under active purgation. I wish to say, however, that I am in favor of operating upon all cases of appendicitis as soon as the diagnosis can be made.

DR. P. GUNTERMANN: As to the rapid formation of pus, and the rapid formation of other tissues: The remarks made by previous speakers recall to my mind a case which is of interest in this connection. Mrs. G. while working about the room at eleven o'clock in the morning had a rupture. At five o'clock in the afternoon she was seen by Dr. Satterwhite and myself, and we advised immediate operation, which was refused. In a few minutes the patient was dead. The woman was ruptured at eleven o'clock in the morning; we urged operation at five o'clock in the afternoon; not being allowed to do so, she died shortly afterwards. A post mortem was held, and we found a small rupture and complete organization. It shows that in this short time complete organization took place. If organization of lymph thrown out can take place so promptly, why may not pus be formed as rapidly as has been stated?

In the case above reported the ring was completely and solidly closed.

